# Supplementary material for: Unveiling Ecological and Genetic Novelty within Lytic and Lysogenic Viral Communities of Hot Spring Phototrophic Microbial Mats
Source: Microbiol Spectr. 2021 Nov 17;9(3):e00694-21. doi: 10.1128/Spectrum.00694-21 (PMC8597652; doi:10.1128/Spectrum.00694-21)
Supplement: SUPPLEMENTAL FILE 1 — Supplemental material. Download SPECTRUM00694-21_Supp_1_seq8.pdf, PDF file, 2.1 MB [file spectrum00694-21_supp_1_seq8.pdf]

Supplementary Material

Unveiling ecological and genetic novelty within lytic and lysogenic viral communities of hot spring phototrophic microbial mats.

Sergio Guajardo-Leiva, Fernando Santos, Oscar Salgado, Christophe Regeard, Laurent Quillet, and Beatriz Díez*.

*** Correspondence:** Beatriz Díez: bdiez@bio.puc.cl

1 Supplementary Figures and Tables

1.1 Supplementary Figures

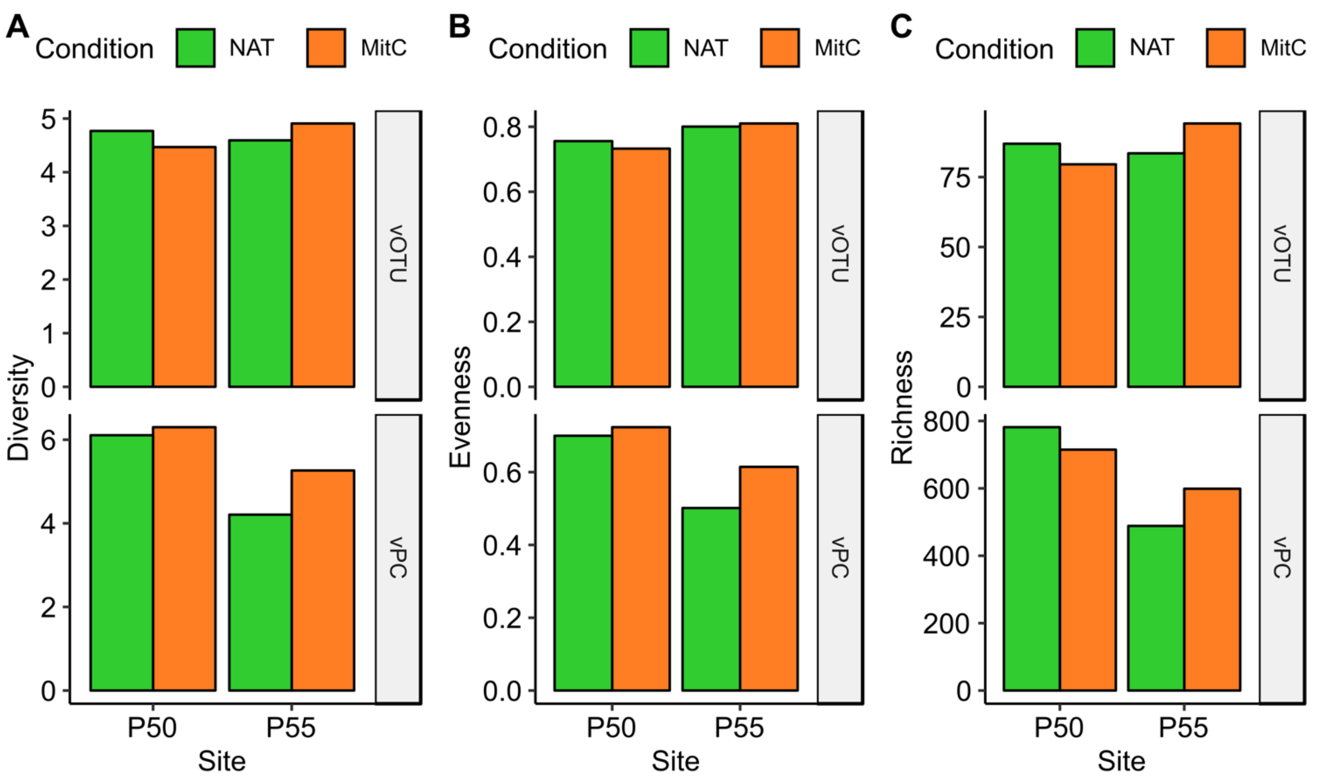

Supplementary Figure S1. Alpha diversity of Porcelana hot spring natural and mitomycin C induced communities at two sites. vPCs and vOTUs normalized counts for each sample were used to calculate A) Shannon's diversity, B) Pielou's evenness and C) Species Richness.

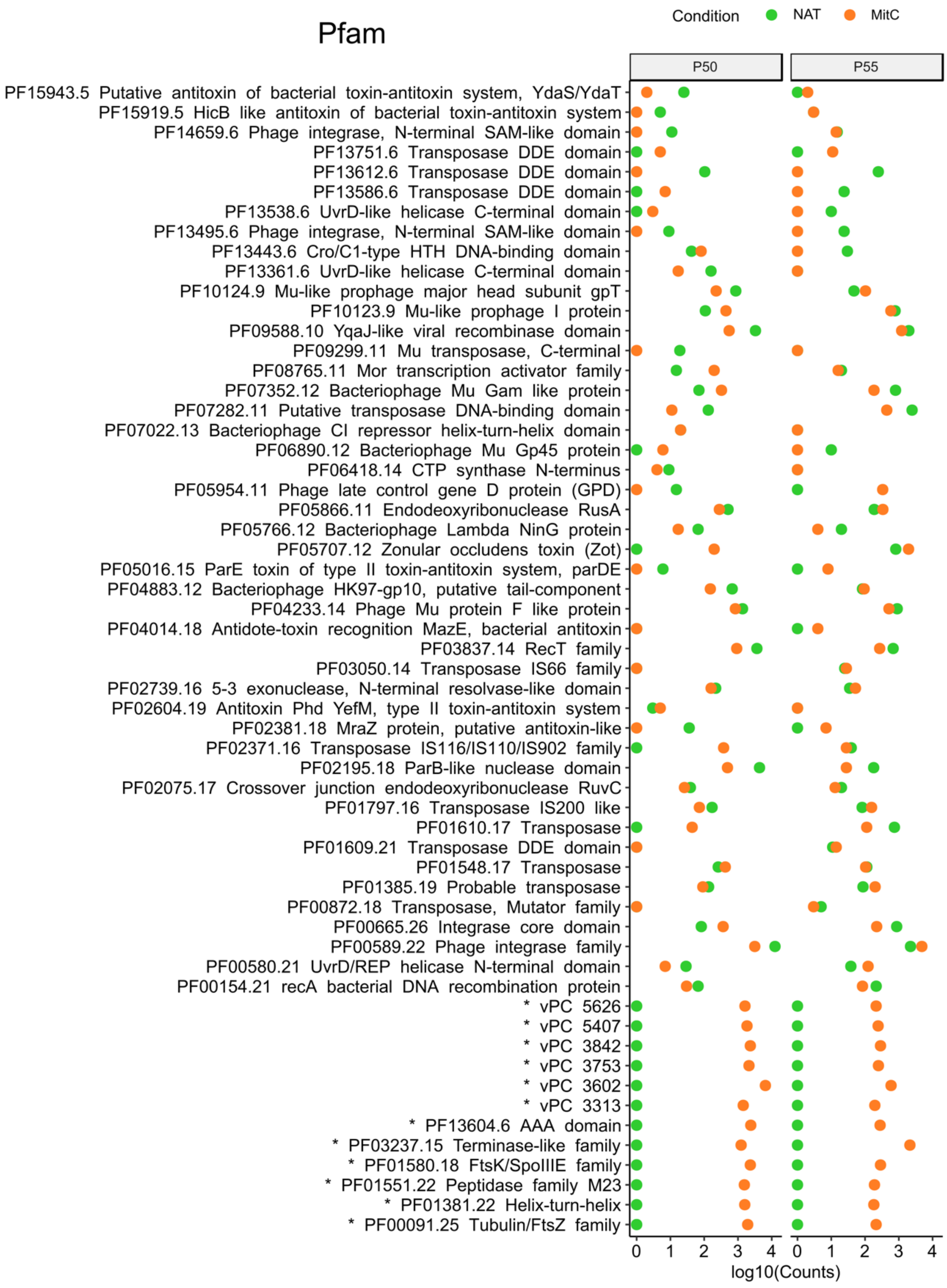

Supplementary Figure S2. Relative abundance of lysogenic related vPCs, obtained from Pfam annotation or differential abundance analyses. Viral PCs obtained by the differential abundance analyses are marked by an asterisk symbol (*).

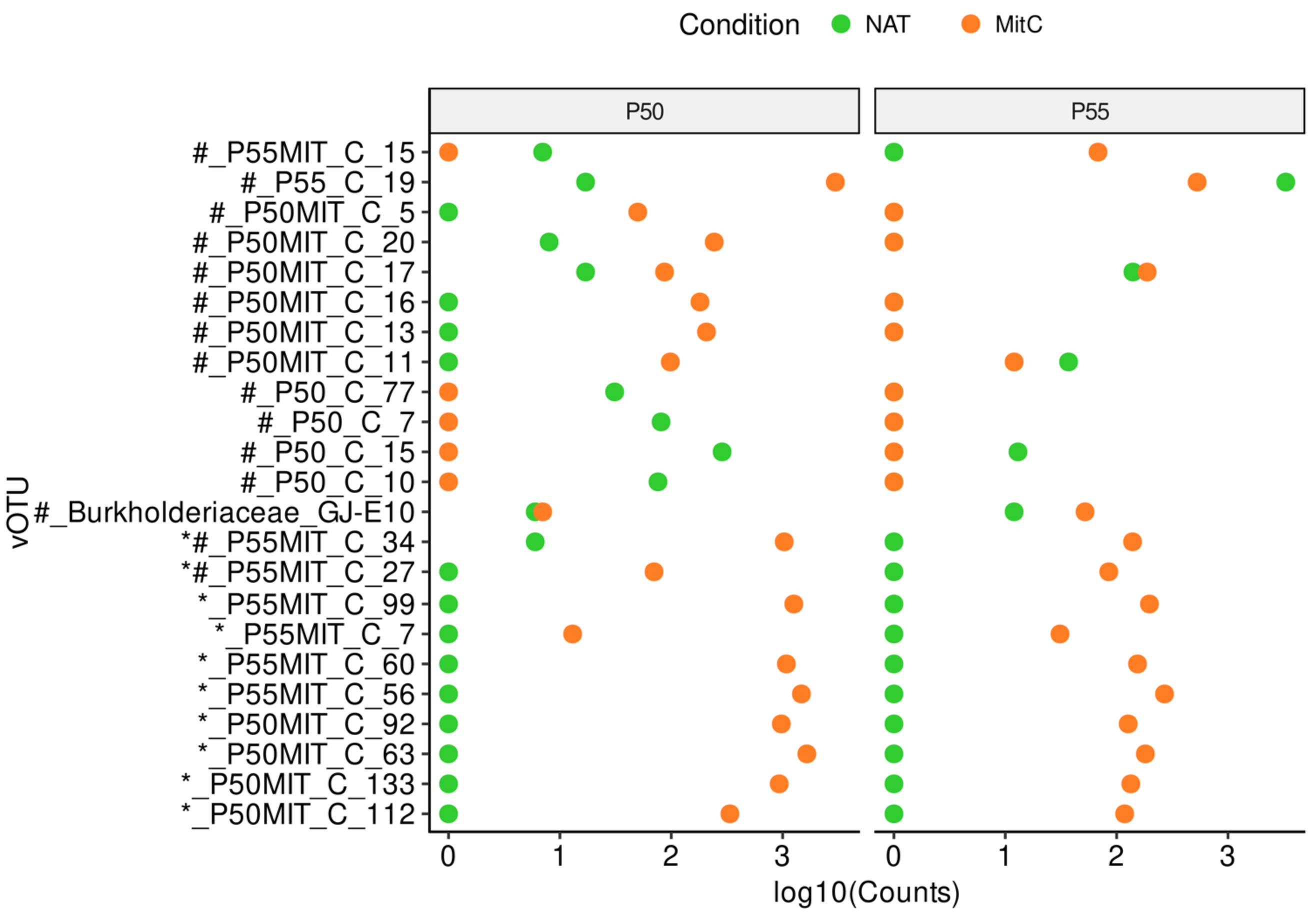

Supplementary Figure S3. Relative abundances of lysogenic vOTUs, obtained from PHASTER annotation or differential abundance analyses. Viral OTUs obtained by PHASTER analyses are marked by a hash symbol (#). vOTUs obtained by the differential abundance analyses are marked by an asterisk symbol (*). vOTUs obtained by both techniques are marked by a hash-asterisk symbol (#*).

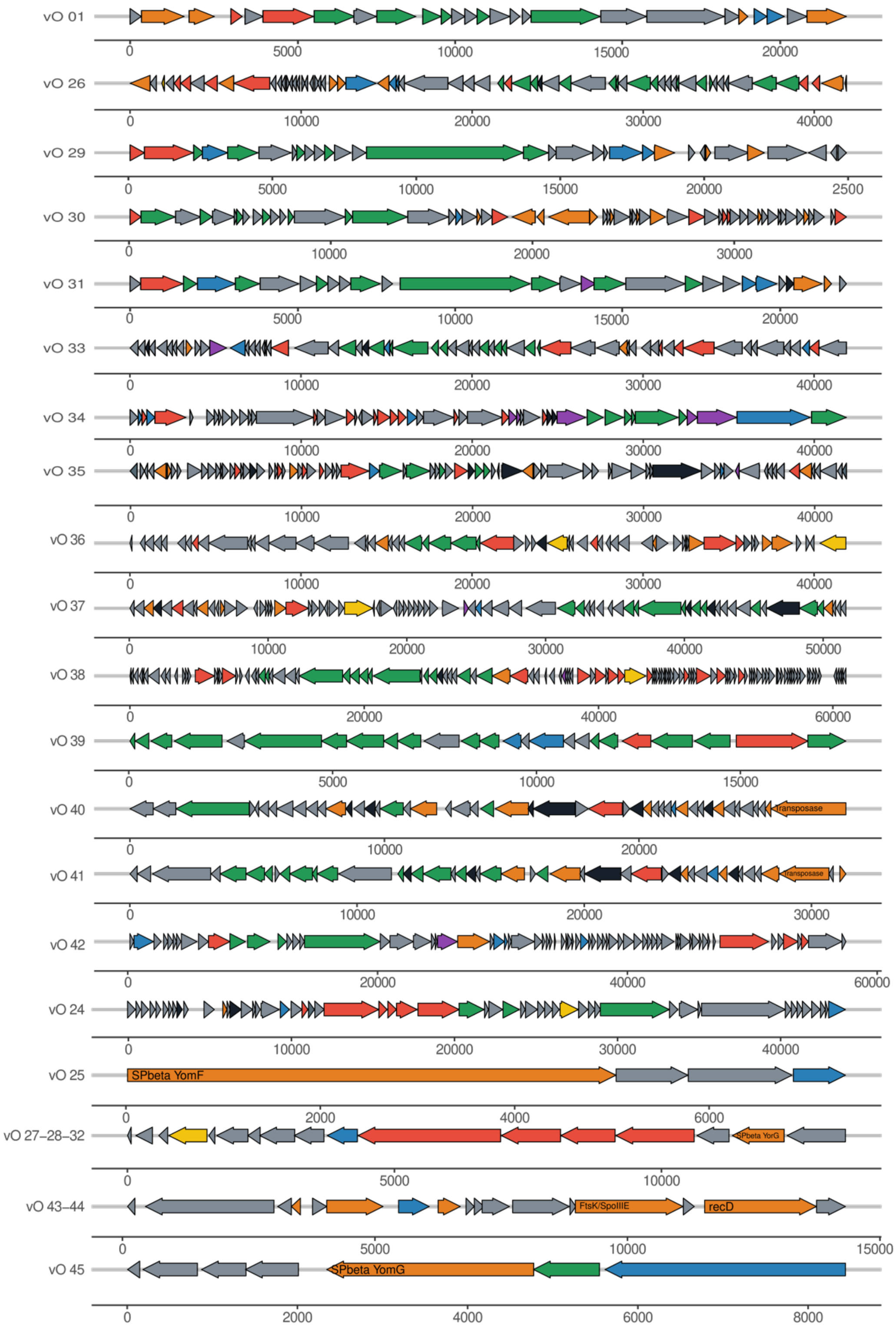

Supplementary Figure S4. Gene map of the lysogenic vOTUs. Arrows represent ORFs and color the functional annotation. ORFs associated with functions or proteins from lysogenic viruses appear in orange.

1.2 Supplementary Tables.

Supplementary Table S1. Summary information about sequencing depth, quality filtering, read mapping and assembly of hot springs viral metagenomes. (M): Millions

Sample	Raw sequences (M)	Sequences (M) after quality filter	Bases (M) after quality filter	% of 16S rRNA sequences	% of NR aligned sequences	% of recruited sequences in PCs	% of recruited sequences in vOTUs
P50NAT	4.13	3.69	859.60	0.2	8.68	37.1	43.9
P55NAT	3.01	2.76	655.01	0.07	5.14	52.9	24.6
P50MitC	8.67	7.88	1811.56	0.07	10.81	26.5	39.2
P55MitC	6.91	6.30	1456.28	0.07	7.01	47.3	36.2

(M): Millions

Supplementary Table S2. Relative abundances of PCs with Pfam annotation. Counts from protein clusters with the same Pfam accession were summed in each sample. Only functions with relative abundances above 0.1% of the total PCs counts are showed.

Pfam	Accession	P50CTRL	P55CTRL	P50MitC	P55MitC
Phage integrase family	PF00589.22	12469	2217	3140	4801
Terminase RNaseH-like domain	PF17289.2	11431	3577	3792	1808
DNA polymerase family A	PF00476.20	5189	2139	3210	6253
Domain of unknown function (DUF4774)	PF15999.5	690	417	1154	13290
Peptidase family M23	PF01551.22	5703	3044	2933	1638
Replication initiation factor	PF02486.19	12055	146	452	226
Bacteriophage head to tail connecting protein	PF12236.8	5668	2586	1388	1131
dUTPase	PF00692.19	4341	1516	1175	2663
N-acetylmuramoyl-L-alanine amidase	PF01510.25	4157	274	1622	3371
Terminase-like family	PF03237.15	858	1408	1604	5343
Poxvirus A32 protein	PF04665.12	431	2477	0	6178
NlpC/P60 family	PF00877.19	49	0	8723	9
Tail tubular protein	PF17212.3	5281	1690	1171	475
Phage portal protein	PF04860.12	5101	257	1800	310
DnaB-like helicase C terminal domain	PF03796.15	4765	97	2347	135
YqaJ-like viral recombinase domain	PF09588.10	3293	1979	547	1212
Phage capsid family	PF05065.13	5042	177	1535	275
Protein of unknown function (DUF1071)	PF06378.11	3792	1760	989	442
N-acetylmuramoyl-L-alanine amidase	PF01520.18	4039	1362	1034	463
Phage terminase large subunit (GpA)	PF05876.12	4455	1064	972	389
Phage-related minor tail protein	PF10145.9	460	1131	763	4316
Thymidylate kinase	PF02223.17	3377	1184	995	415
FtsK/SpoIIIE family	PF01580.18	235	83	2505	3088
Ribonucleotide reductase, barrel domain	PF02867.15	537	207	502	4415
Bacteriophage T4-like capsid assembly protein (Gp20)	PF07230.11	192	149	305	4930
RecT family	PF03837.14	3633	678	917	270
Helix-turn-helix domain	PF12728.7	3923	210	993	156
Glycosyl transferases group 1	PF00534.20	180	170	274	4496
ParB-like nuclease domain	PF02195.18	4339	179	488	27
Major capsid protein Gp23	PF07068.11	148	136	273	4447
Prohead core protein serine protease	PF03420.13	171	120	308	4362

Caudovirus prohead serine protease	PF04586.17	3412	71	1263	213
Protein of unknown function (DUF3987)	PF13148.6	4131	64	439	128
VRR-NUC domain	PF08774.11	2876	161	1437	189
AAA domain	PF13481.6	3042	122	1239	212
Calcineurin-like phosphoesterase	PF00149.28	827	912	1810	1049
Prophage endopeptidase tail	PF06605.11	89	100	1168	3147
Bacterial regulatory protein, Fis family	PF02954.19	3106	55	1104	126
Phage tail protein	PF05709.11	146	120	241	3838
Phage portal protein, lambda family	PF05136.13	3938	0	320	5
Concanavalin A-like lectin/glucanases superfamily	PF13385.6	225	158	228	3429
PD-(D/E)XK nuclease superfamily	PF12705.7	575	628	1587	1128
VirE N-terminal domain	PF08800.10	118	1997	436	1092
Phage Mu protein F like protein	PF04233.14	1364	895	836	510
Putative phage serine protease XkdF	PF14550.6	583	1674	345	940
Toprim-like	PF13155.6	127	91	192	2876
Uncharacterized conserved protein (DUF2190)	PF09956.9	2796	40	285	100
Bacteriophage holin family	PF05105.12	2235	33	845	87
AAA domain (dynein-related subfamily)	PF07728.14	2582	157	270	100
Putative transposase DNA-binding domain	PF07282.11	131	2468	10	439
Meiotically up-regulated gene 113	PF13455.6	176	89	160	2602
Helix-turn-helix	PF01381.22	576	180	1854	375
Zonular occludens toxin (Zot)	PF05707.12	0	811	196	1939
Trypsin-like peptidase domain	PF13365.6	2573	33	248	65
Kelch motif	PF13964.6	74	936	153	1613
Protein of unknown function (DUF935)	PF06074.12	219	1040	576	933
AAA domain	PF13604.6	0	6	2392	278
Glycosyl hydrolase 108	PF05838.12	493	661	1425	39
VWA-like domain (DUF2201)	PF09967.9	2380	0	175	0
Helix-turn-helix domain	PF13560.6	1961	19	461	49
Phage terminase large subunit	PF04466.13	794	306	727	596
Reverse transcriptase (RNA-dependent DNA polymerase)	PF00078.27	2083	0	315	0
SNF2 family N-terminal domain	PF00176.23	258	393	1353	391
CHC2 zinc finger	PF01807.20	1846	97	289	57
Tubulin/FtsZ family, GTPase domain	PF00091.25	0	0	1936	212
Large polyvalent protein associated domain 38	PF18857.1	127	798	116	1106
Helicase conserved C-terminal domain	PF00271.31	92	1117	0	766
Baseplate J-like protein	PF04865.14	62	1098	406	380
Putative amidoligase enzyme	PF12224.8	257	579	262	847
Mu-like prophage I protein	PF10123.9	107	775	440	580
AAA domain	PF13401.6	240	827	509	221
DNA methylase	PF01555.18	1244	133	93	263
Mitochondrial genome maintenance MGM101	PF06420.12	291	562	101	763
Helix-turn-helix domain	PF13730.6	311	535	91	778
P22 coat protein - gene protein 5	PF11651.8	1139	112	373	37
Protein of unknown function (DUF2800)	PF10926.8	270	83	1255	17
Phage virion morphogenesis family	PF05069.13	99	61	1306	77
Integrase core domain	PF00665.26	81	864	362	220
N-terminal phage replisome organiser (Phage rep org N)	PF09681.10	93	0	1426	0
Phage portal protein, SPP1 Gp6-like	PF05133.14	880	139	320	162
Protein of unknown function (DUF2815)	PF10991.8	103	46	1255	4
Chaperonin 10 Kd subunit	PF00166.21	1112	46	181	56
Bacteriophage Mu Gam like protein	PF07352.12	69	798	324	183

Helix-turn-helix domain	PF12844.7	96	784	292	197
Endodeoxyribonuclease RusA	PF05866.11	508	186	280	336
C-5 cytosine-specific DNA methylase	PF00145.17	1034	116	95	30
Mu-like prophage major head subunit gpT	PF10124.9	861	46	225	102
HNH endonuclease	PF01844.23	185	564	290	183
DNA primase catalytic core, N-terminal domain	PF08275.11	0	0	1034	130
Protein of unknown function (DUF1320)	PF07030.12	167	417	172	405
DNA polymerase type B, organellar and viral	PF03175.13	89	370	36	596
Protein of unknown function (DUF1018)	PF06252.12	45	602	250	160
D12 class N6 adenine-specific DNA methyltransferase	PF02086.15	441	227	105	281
Capsid protein (F protein)	PF02305.17	0	107	464	482
Protein of unknown function (DUF3168)	PF11367.8	749	42	125	125
D-alanyl-D-alanine carboxypeptidase	PF13539.6	66	4	946	10
Bacteriophage HK97-gp10, putative tail-component	PF04883.12	673	83	151	93
3D domain	PF06725.11	0	0	889	107
ASCH domain	PF04266.14	880	30	71	5
Bacterial dnaA protein helix-turn-helix	PF08299.11	255	267	447	17
Putative metallopeptidase domain	PF13203.6	870	0	101	3
Phage major capsid protein E	PF03864.15	17	181	391	374
Phage Tail Collar Domain	PF07484.12	351	131	372	101
Polysaccharide deacetylase	PF01522.21	729	8	204	0
Transposase	PF01548.17	259	112	420	103
Transposase	PF01610.17	0	739	43	111
Family of unknown function (DUF5309)	PF17236.2	558	0	169	134
Bacterial regulatory protein, arsR family	PF01022.20	25	518	195	103
Putative bacterial sensory transduction regulator	PF10722.9	746	16	63	14
Replication initiation and membrane attachment	PF07261.11	673	14	132	15
DNA N-6-adenine-methyltransferase (Dam)	PF05869.11	50	275	5	501
Type III restriction enzyme, res subunit	PF04851.15	188	193	16	413
Calcineurin-like phosphoesterase superfamily domain	PF12850.7	132	182	26	455
Phage lysozyme	PF00959.19	80	56	504	109
ERF superfamily	PF04404.12	483	27	215	13
Phage P22-like portal protein	PF16510.5	695	11	21	3
Terminase small subunit	PF03592.16	176	153	180	213
Rad52/22 family double-strand break repair protein	PF04098.15	81	191	67	369
Phage head-tail joining protein	PF05521.11	499	43	125	34
CHAP domain	PF05257.16	622	7	60	7
Anaerobic ribonucleoside-triphosphate reductase	PF13597.6	28	366	176	107
P22 tail accessory factor	PF11650.8	342	64	185	62
Peptidase S24-like	PF00717.23	54	93	418	78
Protein of unknwon function (DUF3310)	PF11753.8	18	20	562	0
Putative phage tail protein	PF13550.6	71	130	39	344
Uracil DNA glycosylase superfamily	PF03167.19	221	100	143	117
Clp protease	PF00574.23	220	248	36	63
N-acetylmuramidase	PF11860.8	148	88	161	152
Peptidase C26	PF07722.13	123	68	273	83
Glutamine amidotransferase class-I	PF00117.28	64	60	228	189
Primase C terminal 1 (PriCT-1)	PF08708.11	525	0	0	0
Methyltransferase domain	PF08241.12	462	12	38	13
Glycosyltransferase family 29 (sialyltransferase)	PF00777.18	475	0	40	0
Probable transposase	PF01385.19	134	86	90	198
Ankyrin repeats (3 copies)	PF12796.7	98	64	0	343

Supplementary Table S3. Results of two-way Brunner-Dette-Munk test of Porcelana vPCs and vOTUs abundances from two sites and mitomycin C induced and natural samples.

Factor	df1	df2	F*	P (F > F*)
**vPCs**				
Condition	1	37083.31	28.31	1.04 x10-07
Site	1	37083.31	801.66	1.70x10-174
Condition:Site	1	37083.31	214.18	2.30x10-48
**vOTUs**				
Condition	1	2948.89	0.45	0.5
Site	1	2948.89	21.28	4.14x10-06
Condition:Site	1	2948.89	27.61	1.59x10-07

Supplementary Table S4: Results of Wilcoxon pairwise comparisons rank sum test of Porcelana vPCs and vOTUs abundances from two sites and mitomycin C induced and natural samples.

Groups	P-value
**vPCs**	
P50MitC:P50NAT	3.22x10-51
P55NAT:P50NAT	5.36x10-185
P55NAT:P50MitC	6.14x10-61
P55MitC:P50NAT	7.18x10-115
P55MitC:P50MitC	2.64x10-25
P55MitC:P55NAT	1.89x10-12
**vOTUs**	
P50NAT:P50MitC	0.0016
P50NAT:P55MitC	0.0454
P55NAT:P50MitC	0.0016
P55NAT:P55MitC	0.0454

Supplementary Table S5. Exclusive PCs and vOTUs from induced communities. Viral OTUs in red bold letters were detected as prophage sequences by PHASTER analyses.

MitC exclusive PCs
3D_domain
AAA_ATPase_domain
AIR_synthase_related_protein,_C-terminal_domain
Anti-sigma-28_factor,_FlgM
Antidote-toxin_recognition_MazE,_bacterial_antitoxin
ATP_phosphoribosyltransferase
Bacterial_regulatory_proteins,_lacI_family
Bacterial_SH3_domain
BtpA_family
C-terminal_AAA-associated_domain
Cation_efflux_family
chorismate_binding_enzyme
DNA_polymerase_III_beta_subunit,_central_domain
DNA_primase_catalytic_core,_N-terminal_domain
Domain_of_unknown_function_(DUF1854)
DsrE/DsrF-like_family
Endonuclease_NucS
FabA-like_domain
Fatty_acid_desaturase
Ferrous_iron_transport_protein_B
Flavodoxin-like_fold
FMN-binding_domain
Formiminotransferase_domain,_N-terminal_subdomain
Glutathione_peroxidase
Glycoprotease_family
Glycosyl_hydrolase_catalytic_core
Glycosyl_hydrolase_family_57
Glycosyl_hydrolase_family_65_central_catalytic_domain
Gram-negative_bacterial_TonB_protein_C-terminal
HD_domain
HEPN_domain
HhH-GPD_superfamily_base_excision_DNA_repair_protein
Iron_dependent_repressor,_metal_binding_and_dimerisation_domain
Iron-sulfur_cluster_assembly_protein
Metallo-beta-lactamase_superfamily
MGS-like_domain
NAD(P)H-binding
Nickel-dependent_hydrogenase
NifU-like_N_terminal_domain
NusB_family
OmpA_family
Outer_membrane_efflux_protein
Oxygen_tolerance
Papain_family_cysteine_protease
Pectate_lyase_superfamily_protein
Peptidase_M16_inactive_domain
Photosynthesis_system_II_assembly_factor_YCF48
PHP_domain
Protein_of_unknown_function_(DUF2764)
Protein_of_unknown_function_(DUF739)
PTS_system_glucitol/sorbitol-specific_IIA_component
Putative_restriction_endonuclease
RadC-like_JAB_domain
Ribose/Galactose_Isomerase
Ribosomal_protein_L10
Ribosome_recycling_factor
RNA_methyltransferase
RNA_recognition_motif._(a.k.a._RRM,_RBD,_or_RNP_domain)
RNase_H
Sir2_family
Stage_II_sporulation_protein_E_(SpoIIE)
Tetratricopeptide_repeat

MitC exclusive vOTUs
P50MIT_C_1
P50MIT_C_103
P50MIT_C_104
P50MIT_C_108
P50MIT_C_112
P50MIT_C_113
P50MIT_C_118
P50MIT_C_123
P50MIT_C_127
P50MIT_C_129
**#P50MIT_C_13**
P50MIT_C_132
P50MIT_C_133
P50MIT_C_134
P50MIT_C_136
P50MIT_C_138
P50MIT_C_141
P50MIT_C_144
P50MIT_C_149
P50MIT_C_153
P50MIT_C_16
P50MIT_C_162
P50MIT_C_165
P50MIT_C_167
P50MIT_C_168
P50MIT_C_170
P50MIT_C_175
P50MIT_C_176
P50MIT_C_179
P50MIT_C_189
P50MIT_C_194
P50MIT_C_195
P50MIT_C_30
P50MIT_C_39
P50MIT_C_40
P50MIT_C_47
**#P50MIT_C_5**
P50MIT_C_59
P50MIT_C_63
P50MIT_C_74
P50MIT_C_81
P50MIT_C_92
P55MIT_C_100
P55MIT_C_101
P55MIT_C_102
P55MIT_C_103
P55MIT_C_106
P55MIT_C_107
P55MIT_C_108
P55MIT_C_109
P55MIT_C_115
P55MIT_C_116
P55MIT_C_117
P55MIT_C_119
P55MIT_C_121
P55MIT_C_123
P55MIT_C_126
P55MIT_C_128
P55MIT_C_129
P55MIT_C_132
P55MIT_C_135
P55MIT_C_137

Toprim_domain
Transglycosylase
TrkA-N_domain
tRNA_synthetases_class_II_core_domain_(F)
Tubulin/FtsZ_family,_GTPase_domain
ubiE/COQ5_methyltransferase_family

P55MIT_C_138
P55MIT_C_141
P55MIT_C_142
P55MIT_C_145
P55MIT_C_146
P55MIT_C_149
P55MIT_C_151
P55MIT_C_152
P55MIT_C_154
P55MIT_C_155
P55MIT_C_157
P55MIT_C_158
P55MIT_C_160
P55MIT_C_162
P55MIT_C_165
P55MIT_C_170
P55MIT_C_171
P55MIT_C_172
P55MIT_C_173
P55MIT_C_174
P55MIT_C_175
P55MIT_C_177
P55MIT_C_180
P55MIT_C_182
P55MIT_C_183
P55MIT_C_185
P55MIT_C_186
P55MIT_C_19
P55MIT_C_190
P55MIT_C_191
P55MIT_C_193
P55MIT_C_194
P55MIT_C_196
P55MIT_C_201
P55MIT_C_202
P55MIT_C_24
P55MIT_C_26
P55MIT_C_27
P55MIT_C_29
P55MIT_C_45
P55MIT_C_46
P55MIT_C_5
P55MIT_C_50
P55MIT_C_54
P55MIT_C_56
P55MIT_C_57
P55MIT_C_60
P55MIT_C_62
P55MIT_C_63
P55MIT_C_64
P55MIT_C_7
P55MIT_C_74
P55MIT_C_84
P55MIT_C_85
P55MIT_C_86
P55MIT_C_87
P55MIT_C_88
P55MIT_C_89
P55MIT_C_92
P55MIT_C_99

Supplementary Table S6. Relative abundances of singletons with Pfam annotation. Counts from singletons with the same Pfam accession were summed in each sample. Only functions with relative abundances above 1 % of the total singletons counts are showed.

Pfam	Fuction	P50NAT	P55NAT	P50MitC	P55MitC
PF04233.14	Phage Mu protein F like protein	2180	3039	21294	17916
PF15999.5	Domain of unknown function (DUF4774)	1287	1337	0	37022
PF06074.12	Protein of unknown function (DUF935)	2890	2957	16770	16195
PF11367.8	Protein of unknown function (DUF3168)	4510	478	168	27124
PF17288.2	Terminase RNAseH like domain	476	29956	17231	9508
PF11697.8	Protein of unknown function (DUF3293)	104	7686	24282	170
PF00574.23	Clp protease	8405	1162	21257	1434
PF00176.23	SNF2 family N-terminal domain	12054	5244	1785	19319
PF12965.7	Domain of unknown function (DUF3854)	3336	0	0	20890
PF02811.19	Bacterial DNA polymerase III alpha NTPase domain	3006	763	16940	3636
PF12705.7	AAA domain	19599	10236	7445	10589
PF13857.6	Ankyrin repeats (3 copies)	1455	9626	5661	11874
PF03864.15	Phage major capsid protein E	2284	0	16459	835
PF10145.9	Phage-related minor tail protein	10121	6077	9702	7573
PF13524.6	Glycosyl transferases group 1	5127	5739	5921	10999
PF04860.12	Phage portal protein	14364	6714	10711	5959
PF03796.15	DnaB-like helicase C terminal domain	5620	82780	2900	12428
PF00415.18	Regulator of chromosome condensation (RCC1) repeat	197	470	14109	53
PF13529.6	Peptidase_C39 like family	53	1238	13034	544
PF04984.14	Phage tail sheath protein subtilisin-like domain	1569	247	200	12784
PF00589.22	Phage integrase family	9459	21631	3369	9430
PF13884.6	Chaperone of endosialidase	743	15556	265	12265
PF03354.15	Phage Terminase	14453	4976	4828	6906
PF02924.14	Bacteriophage lambda head decoration protein D	250	150	11605	39
PF10127.9	RNA repair pathway DNA polymerase beta family	85	311	11297	322
PF01464.20	Transglycosylase SLT domain	6658	3818	1987	9518
PF00004.29	ATPase family associated with various cellular activities (AAA)	4583	1329	9692	1351
PF05065.13	Phage capsid family	14541	5800	6135	4383
PF02498.17	BRO family, N-terminal domain	5043	0	10442	0
PF06252.12	Protein of unknown function (DUF1018)	30	145	9724	285
PF01555.18	DNA methylase	10160	3355	5175	3132
PF13395.6	HNH endonuclease	12282	2287	5705	2571
PF00476.20	DNA polymerase family A	17946	5066	3086	2823
PF02086.15	D12 class N6 adenine-specific DNA methyltransferase	3204	30485	801	4699
PF13148.6	Protein of unknown function (DUF3987)	1267	14956	470	3947
PF03237.15	Terminase-like family	14054	56976	1886	1922
PF12705.7	PD-(D/E)XK nuclease superfamily	19332	3427	1650	1856
PF09250.11	Bifunctional DNA primase/polymerase, N-terminal	2050	12742	559	1625
PF01935.17	Helicase HerA, central domain	458	89765	267	1090
PF05069.13	Phage virion morphogenesis family	944	16075	162	597
PF05272.11	Virulence-associated protein E	664	10880	498	90

Supplementary Table S7. Genome annotations.

Genome	gene	start	end
vO_01	hp	325	669
vO_01	Transposase	675	1988
vO_01	Transposase	2139	2918
vO_01	HNH endonuclease	3426	3773
vO_01	hp	3861	4421
vO_01	Terminase	4411	5964
vO_01	Portal	5987	7243
vO_01	hp	7209	7901
vO_01	Capsid	7914	9116
vO_01	Head completion	9328	9900
vO_01	Head completion	9897	10223
vO_01	hp	10216	10623
vO_01	hp	10620	10997
vO_01	Tail	11000	11383
vO_01	hp	11396	12010
vO_01	hp	12017	12316
vO_01	hp	12391	12654
vO_01	Tape measure	12661	14790
vO_01	hp	14805	16208
vO_01	hp	16221	18605
vO_01	hp	18624	19085
vO_01	Transposase	19051	19329
vO_01	Holin	19523	19918
vO_01	N-acetylmuramoyl-L-alanine amidase	19921	20454
vO_01	hp	20546	21154
vO_01	Transposase	21147	22361
vO_02-04	hp	316	795
vO_02-04	hp	1345	1986
vO_02-04	Spbeta YonF (terminase motif)	1986	3368
vO_02-04	hp	3380	5269
vO_02-04	hp	5308	6525
vO_02-04	hp	6522	6764
vO_02-04	hp	6775	7320
vO_02-04	hp	7332	8756
vO_02-04	hp	8782	9210
vO_02-04	hp	9214	9699
vO_02-04	hp	9689	9940
vO_02-04	hp	9937	10335
vO_02-04	hp	10328	11053
vO_02-04	hp	11053	11565
vO_02-04	hp	11663	12934
vO_02-04	hp	12949	13743
VO_02-04	hp	13802	14272
vO_02-04	hp	14471	15226
vO_02-04	hp	15325	17043
vO_02-04	hp	17056	17901
vO_02-04	hp	17903	18211
vO_02-04	hp	18208	19587
vO_02-04	hp	19584	20255
vO_02-04	Baseplate J	20257	20640
vO_02-04	hp	20637	21803
vO_02-04	hp	21806	22666
vO_02-04	hp	22663	23913
vO_02-04	hp	23913	24179
vO_02-04	lipase	24163	24423
vO_02-04	hp	24407	24991
vO_02-04	N-acetylmuramoyl-L-alanine amidase	24981	25463
vO_02-04	hp	25460	26053
vO_02-04	hp	26050	26265
vO_02-04	hp	26316	26555
vO_02-04	hp	26533	26703
vO_02-04	DNA-Methyltransferase	26678	27103
vO_02-04	hp	27203	28315
vO_02-04	hp	28312	28551
vO_02-04	hp	28561	28890

vO_02-04	hp	29355	29600
vO_02-04	hp	29609	29941
vO_02-04	hp	30086	31255
vO_02-04	DEAD box helicase	31279	31590
vO_02-04	hp	31583	32887
vO_02-04	DNA pol	32884	33270
vO_02-04	ATP-dependent DNA Helicase PcrA	33251	35239
vO_02-04	hp	35217	36710
vO_02-04	hp	36714	37388
vO_02-04	hp	37354	37935
vO_02-04	hp	37916	39556
vO_02-04	DUF1351	39621	40499
vO_02-04	hp	40502	41395
vO_02-04	hp	41388	42008
vO_02-04	hp	42005	42835
vO_02-04	hp	42836	43279
vO_02-04	hp	43251	43676
vO_02-04	hp	43782	44078
vO_02-04	hp	44072	44197
vO_02-04	hp	44194	44784
vO_02-04	hp	44796	45041
vO_02-04	hp	45346	45765
vO_02-04	DNA repair RadC	45805	46416
vO_02-04	hp	47057	47815
vO_02-04	hp	47812	48090
vO_02-04	hp	48154	48606
vO_05	hp	3	344
vO_05	hp	328	534
vO_05	hp	531	956
vO_05	hp	940	1155
vO_05	hp	1155	1472
vO_05	hp	1469	1747
vO_05	DUF3854	1752	4601
vO_05	hp	4648	4899
vO_05	hp	4878	5426
vO_05	hp	5426	5698
vO_05	hp	5698	5946
vO_05	hp	6104	6232
vO_06	hp	1	720
vO_06	hp	708	1649
vO_06	RecT family	1761	2486
vO_06	exodeoxyribonuclease	2483	3085
vO_06	hp	3064	3474
vO_06	DUF3987	3471	5654
vO_06	hp	5781	6002
vO_06	Helix-turn-helix domain	6007	6588
vO_06	Trypsin-like peptidase domain	6940	7977
vO_06	hp	7977	8156
vO_06	hp	8198	9937
vO_06	hp	9934	10791
vO_06	hp	10810	12642
vO_06	hp	12935	13297
vO_06	hp	13284	13712
vO_06	hp	13737	14210
vO_06	hp	14237	14701
vO_06	hp	14705	15076
vO_06	DUF2190	15098	15430
vO_06	Portal	15481	19068
vO_06	Terminase large subunit	19307	20113
vO_06	hp	20055	21383
vO_06	Beta-glucosyl-HMC-alpha-glucosyl-transferase	21373	21555
vO_06	hp	21552	22382
vO_06	Nuclease	22393	23665
vO_07,10	hp	3	425
vO_07,10	hp	425	1219
vO_07,10	hp	1216	1560
vO_07,10	hp	1563	2099
vO_07,10	hp	2096	2578
vO_07,10	hp	2735	3586

vO_07,10	hp	3639	4208
vO_07,10	hp	4209	4745
vO_07,10	hp	4742	5197
vO_07,10	hp	5208	5387
vO_08,09,11,13	hp	332	628
vO_08,09,11,13	hp	646	1197
vO_08,09,11,13	hp	1216	1704
vO_08,09,11,13	hp	1744	2097
vO_08,09,11,13	Terminase large subunit	2101	3810
vO_08,09,11,13	Portal	3810	5855
vO_08,09,11,13	Scaffold	5852	6766
vO_08,09,11,13	Minor capsid	6786	7820
vO_08,09,11,13	hp	7903	8124
vO_08,09,11,13	Tail tubular	8121	8813
vO_08,09,11,13	Tail tubular B	8815	11997
vO_08,09,11,13	hp	12014	12283
vO_08,09,11,13	hp	12286	13485
vO_08,09,11,13	Portal	13478	16843
vO_08,09,11,13	PfWMP3_26	16845	20462
vO_08,09,11,13	PfWMP3_25	20468	23809
vO_08,09,11,13	hp	23806	24339
vO_08,09,11,13	Variable tail fiber	24349	25605
vO_08,09,11,13	N-acetylmuramoyl-L-alanine amidase	25810	26370
vO_08,09,11,13	hp	26354	26578
vO_08,09,11,13	hp	26581	26772
vO_08,09,11,13	dCTP deaminase	26778	27362
vO_08,09,11,13	hp	27377	27640
vO_08,09,11,13	SIRV-1 phage type exonuclease	27637	28485
vO_08,09,11,13	hp	28554	28823
vO_08,09,11,13	hp	28835	29446
vO_08,09,11,13	DNA pol	29471	31321
vO_08,09,11,13	hp	31302	31592
vO_08,09,11,13	Primase/Helicase	31643	33880
vO_08,09,11,13	hp	33834	34304
vO_08,09,11,13	hp	34301	34483
vO_08,09,11,13	hp	34616	34750
vO_12	P22 coat	1	648
vO_12	hp	641	1543
vO_12	Portal	1536	3509
vO_12	Terminase large subunit	3506	5152
vO_12	hp	5121	5609
vO_12	hp	5992	6345
vO_12	hp	6563	6832
vO_14,16-17	hp	23	2728
vO_14,16-17	hp	2730	3848
vO_14,16-17	hp	3850	4449
vO_14,16-17	hp	4428	4568
vO_14,16-17	hp	4568	5119
vO_14,16-17	Nuclease	5116	5379
vO_14,16-17	Terminase large subunit	5376	6629
vO_14,16-17	hp	6622	6900
vO_14,16-17	hp	6876	7076
vO_14,16-17	murein DD-endopeptidase	7309	7701
vO_14,16-17	Holin	7661	8134
vO_14,16-17	hp	8131	8436
vO_14,16-17	hp	8433	9686
vO_14,16-17	hp	9821	10177
vO_14,16-17	hp	10177	10743
vO_14,16-17	hp	10748	11215
vO_14,16-17	hp	11215	12993
vO_14,16-17	hp	12990	13343
vO_15	Peptidoglycan DL-endopeptidase CwlO	105	635
vO_15	hp	637	909
vO_15	hp	921	2507
vO_15	Central straight fiber	2504	4660
vO_15	hp	4662	8132
vO_15	hp	8129	8221
vO_15	hp	8446	8838
vO_15	hp	8853	9527

vO_15	hp	9530	9721
vO_15	hp	9734	10168
vO_15	hp	10169	10732
vO_15	hp	10729	11184
vO_15	Major Capsid	11204	12175
vO_15	hp	12191	12550
vO_15	hp	12560	13426
vO_15	Mu-like F	13944	15134
vO_15	Mu-like gp29	15341	16582
vO_15	Terminase large subunit	16579	18123
vO_15	DUF3486	18120	18641
vO_15	hp	18643	18939
vO_15	hp	18936	19289
vO_15	hp	19289	19660
vO_15	hp	19823	20026
vO_15	hp	20037	20678
vO_15	hp	20747	21523
vO_15	hp	21571	22479
vO_15	Repressor LexA	22506	23561
vO_15	hp	23732	23992
vO_15	SPBeta YoqD	23989	24690
vO_15	hp	24687	25061
vO_15	hp	25005	25187
vO_15	hp	25272	25460
vO_15	Transposase	25457	27289
vO_15	ATP-dependent target DNA activator	27305	28195
vO_15	DUF3102	28205	28903
vO_15	hp	28900	29109
vO_15	Mu phage Gam like	29122	29631
vO_15	GemA	29704	30123
vO_15	hp	30120	30299
vO_18-19	Terminase large subunit	3	1037
vO_18-19	Portal	1025	2239
vO_18-19	hp	2236	2415
vO_18-19	hp	2507	3022
vO_18-19	hp	3031	3642
vO_18-19	hp	3828	4307
vO_18-19	hp	4366	6354
vO_18-19	hp	6351	6929
vO_18-19	Major Capsid	6938	8134
vO_18-19	Holin	8333	8794
vO_18-19	hp	8748	9068
vO_18-19	hp	9061	9483
vO_18-19	hp	9505	9948
vO_18-19	hp	9951	10394
vO_18-19	hp	10394	10822
vO_18-19	hp	10989	12521
vO_18-19	hp	12514	14640
vO_18-19	hp	14637	15071
vO_18-19	hp	15071	18343
vO_18-19	hp	18402	18710
vO_18-19	Endolysin	18871	19707
vO_18-19	hp	19741	19959
vO_18-19	hp	20084	20326
vO_18-19	hp	20402	20725
vO_18-19	hp	20959	21297
vO_18-19	hp	21449	21619
vO_18-19	hp	21561	21812
vO_18-19	hp	21873	22046
vO_18-19	hp	22207	22428
vO_18-19	AAA domain	22415	22795
vO_18-19	hp	22776	24029
vO_18-19	Uncharacterized nin region ORF290	24299	24448
vO_18-19	hp	24448	25227
vO_18-19	hp	25461	25889
vO_18-19	hp	25882	26295
vO_18-19	Adenine acetyltransferase mom	26387	26773
vO_18-19	hp	26743	27405
vO_18-19	hp	27492	28020

vO_20	Mu-like prophage I	183	1067
vO_20	Mu-like F	1064	2140
vO_20	DUF935	2127	3575
vO_20	hp	3545	3865
vO_20	hp	3838	4056
vO_20	Terminase large subunit	4082	5032
vO_21	Packaged DNA stabilization gp10	1	492
vO_21	hp	492	1589
vO_21	Packaged DNA stabilization gp10	1592	2404
vO_21	Autotransporter	2401	3180
vO_21	hp	3593	4135
vO_21	hp	4157	5434
vO_21	hp	5434	5508
vO_22	hp	1	96
vO_22	hp	98	583
vO_22	Terminase	567	2279
vO_22	hp	2289	2432
vO_22	Portal	2454	3656
vO_22	hp	3653	4384
vO_22	Capsid	4396	5391
vO_22	Head completion	5406	5924
vO_22	hp	5921	6409
vO_22	hp	6406	6849
vO_22	hp	6825	7034
vO_22	hp	7034	8008
vO_22	hp	8024	8380
vO_22	hp	8329	8619
vO_22	hp	8623	11757
vO_22	hp	11759	15337
vO_22	Sortilin	15349	16476
vO_22	hp	16473	16868
vO_22	hp	16837	17157
vO_22	Peptidoglycan LD-endopeptidase CwlK	17154	17642
vO_22	hp	17635	17898
vO_22	hp	17902	18273
vO_22	hp	18430	19035
vO_22	hp	19123	19392
vO_22	hp	19394	19645
vO_22	hp	19658	20251
vO_22	hp	20322	20522
vO_22	hp	20828	21340
vO_22	hp	21370	21681
vO_22	hp	21669	21935
vO_22	hp	22088	22291
vO_22	hp	22302	22646
vO_22	hp	22633	23076
vO_22	hp	23054	23179
vO_22	Essential recombination function	23191	23847
vO_22	hp	23858	23986
vO_22	hp	23970	24182
vO_22	hp	24179	24298
vO_22	hp	24405	24680
vO_22	Phage replisome organiser	24708	25586
vO_22	hp	25576	25923
vO_22	Endodeoxyribonuclease RusA	25920	26339
vO_22	hp	26320	26469
vO_22	hp	26566	26760
vO_22	HNH endonuclease	26830	27210
vO_23	hp	794	1114
vO_23	hp	2035	2508
vO_23	hp	2589	2903
vO_23	DNA pol	3098	5923
vO_23	hp	5937	6818
vO_23	hp	6858	7103
vO_23	Deoxynucleotide monophosphate kinase	7100	7699
vO_23	hp	7838	8092
vO_23	hp	8079	8765
vO_23	hp	8758	9039
vO_23	Terminase large subunit	9058	10701

vO_23	Portal	10685	12304
vO_23	Prohead core protease	12350	13384
vO_23	Major Capsid	13479	14681
vO_23	hp	14782	15573
vO_23	Glycosyltransferase	15720	16958
vO_23	Ribonucleoside-triphosphate reductase	17058	19052
vO_23	hp	19142	19675
vO_23	hp	19680	20072
vO_23	hp	20089	20673
vO_23	hp	20729	21196
vO_23	hp	21263	21820
vO_23	hp	21853	22359
vO_23	Minor tail	22725	28688
vO_23	Tail	28857	30938
vO_23	hp	30951	31979
vO_23	Concanavalin A-like lectin/glucanases	32048	36316
vO_23	N-acetylmuramoyl-L-alanine amidase	36466	37329
vO_23	hp	37372	37779
vO_23	hp	37766	38191
vO_23	hp	38204	38878
vO_23	hp	39044	39235
vO_23	hp	39235	39834
vO_23	hp	39990	40565
vO_23	hp	40540	40794
vO_23	Helicase	40809	42182
vO_23	hp	42301	43188
vO_23	DNA primase	43213	44313
vO_23	dUTP pyrophosphatase	44555	45052
vO_23	hp	45068	45304
vO_23	hp	45540	46328
vO_23	hp	46478	47572
vO_23	hp	47572	48099
vO_23	hp	48304	48672
vO_23	FtsK/SpoIIIE	48715	50526
vO_23	hp	50706	51029
vO_23	hp	51076	51330
vO_23	hp	51519	52178
vO_23	hp	52245	52454
vO_23	hp	52429	53385
vO_23	Integrase/recombinase XerC	53508	54437
vO_23	hp	54482	54808
vO_23	hp	54956	55936
vO_23	hp	55973	56548
vO_23	hp	56704	57552
vO_23	hp	57599	58351
vO_23	hp	58413	59096
vO_23	hp	59292	59573
vO_23	hp	59643	60671
vO_23	hp	60900	61178
vO_23	hp	61410	62117
vO_23	hp	62356	62727
vO_23	hp	62851	62919
vO_24	hp	1246	1725
vO_24	hp	1763	2035
vO_24	hp	2098	2535
vO_24	hp	2625	2936
vO_24	hp	2986	3282
vO_24	hp	3433	3726
vO_24	hp	3767	3976
vO_24	hp	4021	4251
vO_24	Domain of unknown function (DUF1874)	4274	4600
vO_24	hp	4694	4969
vO_24	hp	5934	6569
vO_24	Sporulation protein D	7089	7346
vO_24	hp	7349	7540
vO_24	DUF4417	7527	8225
vO_24	hp	8218	8919
vO_24	hp	8919	9080
vO_24	hp	9089	9382

vO_24	hp	9437	10546
vO_24	Transglycosylase	10616	11176
vO_24	hp	11277	11948
vO_24	ClpX heat shock protein	11945	12307
vO_24	YspA SLOG family	12342	12707
vO_24	hp	12731	13309
vO_24	Ribonucleoside-diphosphate reductase	13299	16643
vO_24	dUTP pyrophosphatase	16660	17163
vO_24	Deoxynucleotide monophosphate kinase	17211	17738
vO_24	DNA ligase	17742	19004
vO_24	Terminase large subunit	19059	21578
vO_24	Portal protein	21581	23113
vO_24	hp	23141	23377
vO_24	hp	23404	24252
vO_24	Major capsid protein	24274	25278
vO_24	hp	25357	25635
vO_24	hp	25652	26386
vO_24	hp	26400	26816
vO_24	hp	26827	27312
vO_24	hp	27305	27718
vO_24	L-malate glycosyltransferase	27771	28889
vO_24	hp	28910	29473
vO_24	hp	29511	29912
vO_24	hp	29900	30256
vO_24	Tail tape measure protein	30243	34463
vO_24	hp	34484	34999
vO_24	hp	35091	36224
vO_24	hp	36236	36412
vO_24	hp	36450	41588
vO_24	hp	41585	41947
vO_24	hp	41955	42290
vO_24	hp	42305	42631
vO_24	hp	42634	43158
vO_24	hp	43202	43726
vO_24	hp	43743	44042
vO_24	hp	44046	44183
vO_24	Cysteine protease	44242	45276
vO_25	SPbeta YomF	153	5177
vO_25	hp	5180	5917
vO_25	hp	5922	6989
vO_25	Cysteine protease	7003	7539
vO_26	Transposase	249	1358
vO_26	hp	1364	1708
vO_26	DNA methylase	2017	2154
vO_26	hp	2245	2673
vO_26	Endodeoxyribonuclease RusA	2701	3045
vO_26	SPP1 G40P	3062	3613
vO_26	hp	3610	4365
vO_26	Metallo-hydrolase YycJ	4394	5095
vO_26	Recombination protein RecT	5193	6008
vO_26	Exonuclease	6010	8016
vO_26	hp	8082	8354
vO_26	hp	8446	8670
vO_26	hp	8686	8877
vO_26	hp	8946	9098
vO_26	hp	9110	9274
vO_26	hp	9271	9498
vO_26	hp	9524	9673
vO_26	hp	9795	9962
vO_26	hp	9952	10119
vO_26	hp	10244	10432
vO_26	hp	10513	10749
vO_26	hp	10673	10855
vO_26	hp	10873	11109
vO_26	sinR Master regulator for biofilm formation	11326	11772
vO_26	sinR Master regulator for biofilm formation	11783	12217
vO_26	Peptidase M78	12245	13933
vO_26	Site-specific DNA recombinase	14004	14639
vO_26	N-acetylmuramoyl-L-alanine amidase	14642	15037

vO_26	Holin	15040	15195
vO_26	hp	15192	15500
vO_26	hp	15504	17918
vO_26	hp	17920	18759
vO_26	hp	18764	19375
vO_26	hp	19379	20251
vO_26	Tail fiber receptor-binding protein	20691	21011
vO_26	Ribonuclease III	21059	21460
vO_26	Tail fiber protein	21467	22504
vO_26	Baseplate J-like protein	22504	22908
vO_26	DUF2634	22905	23147
vO_26	hp	23144	24094
vO_26	Baseplate hub protein	24091	24756
vO_26	hp	24756	26669
vO_26	Tape measure protein	26860	27261
vO_26	Tail assembly chaperone	27264	27386
vO_26	hp	27393	27866
vO_26	Phage tail tube protein	27873	29171
vO_26	Tail sheath protein	29197	29592
vO_26	hp	29582	29989
vO_26	hp	29986	30321
vO_26	hp	30318	30626
vO_26	Head completion protein	30604	30756
vO_26	hp	30777	31706
vO_26	Minor capsid protein	31710	32285
vO_26	Phage minor structural protein	32469	32630
vO_26	hp	32632	32871
vO_26	hp	32898	33251
vO_26	YjcQ protein	33315	33524
vO_26	hp	33517	34836
vO_26	Minor head protein	34833	36170
vO_26	Portal protein	36180	37427
vO_26	Terminase large subunit	37432	37926
vO_26	Terminase small subunit	38156	38584
vO_26	Transcriptional activator rinA	38682	39887
vO_26	Transposase	39997	40071
vO_27-28-32	hp	3	71
vO_27-28-32	hp	158	469
vO_27-28-32	hp	588	755
vO_27-28-32	DNA-methyltransferase (adenine-specific)	775	1491
vO_27-28-32	hp	1525	1638
vO_27-28-32	hp	1671	2258
vO_27-28-32	hp	2255	2485
vO_27-28-32	hp	2478	3134
vO_27-28-32	hp	3124	3681
vO_27-28-32	L-alanyl-D-glutamate peptidase	3741	4307
vO_27-28-32	DNA polymerase III subunit alpha	4320	6989
vO_27-28-32	Restriction endonuclease	7002	8111
vO_27-28-32	DNA primase	8116	9132
vO_27-28-32	Replicative DNA helicase	9147	10613
vO_27-28-32	hp	10664	11266
vO_27-28-32	SPbeta YorG	11327	12298
vO_27-28-32	hp	12352	13446
vO_29	Terminase small subunit	59	559
vO_29	Terminase	556	2253
vO_29	Portal protein	2266	2619
vO_29	Clp protease	2570	3448
vO_29	Major capsid protein	3448	4497
vO_29	hp	4534	5652
vO_29	hp	5695	5847
vO_29	Head completion protein	5847	6131
vO_29	hp	6128	6460
vO_29	hp	6460	6819
vO_29	Major tail protein	6816	7139
vO_29	hp	7160	7729
vO_29	hp	7783	8252
vO_29	Tape measure protein	8270	13720
vO_29	Phage tail protein	13722	14585
vO_29	hp	14597	14878

vO_29	hp	14856	16121
vO_29	hp	16132	16509
vO_29	hp	16511	16645
vO_29	Holin family	16714	17859
vO_29	N-acetylmuramoyl-L-alanine amidase	17859	18275
vO_29	Putative transcriptional regulator	18272	18976
vO_29	hp	19460	19675
vO_29	hp	19861	20022
vO_29	FtsK/SpoIIIE protein	20056	20226
vO_29	hp	20372	21547
vO_29	Site-specific DNA recombinase	21507	22091
vO_29	HTH_19 domain protein	22216	23559
vO_29	Helix-turn-helix	23618	24238
vO_29	hp	24397	24603
vO_29	hp	24650	24941
vO_30	Phage Terminase	53	601
vO_30	Phage portal protein	594	2291
vO_30	hp	2307	3554
vO_30	Major capsid protein gp17	3526	4134
vO_30	hp	4139	5233
vO_30	hp	5214	5360
vO_30	Head completion protein	5332	5643
vO_30	hp	5643	5972
vO_30	hp	6157	6618
vO_30	Tail fiber protein	6618	7010
vO_30	hp	7014	7430
vO_30	hp	7479	7781
vO_30	Tail tape measure protein	7892	8149
vO_30	hp	8194	10707
vO_30	Tail protein	10720	11067
vO_30	Minor tail protein	11082	13802
vO_30	hp	13817	15835
vO_30	hp	15850	16161
vO_30	N-acetylmuramoyl-L-alanine amidase	16199	16495
vO_30	hp	16515	17231
vO_30	Antitoxin SocA	17244	17423
vO_30	hp	17471	18007
vO_30	micrococcal nuclease	17997	18782
vO_30	Lambda repressor protein cI	18985	20136
vO_30	Site-specific DNA recombinase	20222	20569
vO_30	sinR Master regulator for biofilm formation	20794	22827
vO_30	Repressor protein CI	22833	23225
vO_30	hp	23495	23704
vO_30	hp	23691	23852
vO_30	Phage regulatory protein Rha	23845	24018
vO_30	hp	24041	24808
vO_30	hp	24830	24934
vO_30	hp	24940	25155
vO_30	Bacteriophage Mu Gam like protein	25157	25300
vO_30	hp	25302	25841
vO_30	Anti-repressor	25842	26588
vO_30	hp	26677	27708
vO_30	DNA replication protein DnaC	27752	28555
vO_30	hp	28527	29234
vO_30	hp	29235	29411
vO_30	HNH endonuclease L247	29414	29638
vO_30	single-strand DNA-binding protein	29653	29853
vO_30	hp	29793	30287
vO_30	hp	30277	30663
vO_30	hp	30676	31179
vO_30	Holliday junction resolvase RecU	31176	31355
vO_30	hp	31367	31657
vO_30	hp	31668	32165
vO_30	hp	32179	32334
vO_30	hp	32303	32575
vO_30	hp	32550	33098
vO_30	hp	33101	33433
vO_30	Transcriptional activator rinA	33446	33700
vO_30	hp	33694	33939

vO_30	hp	33944	34465
vO_30	hp	34796	34942
vO_30	HNH endonuclease	34996	35561
vO_31	hp	72	392
vO_31	Phage Terminase	389	1657
vO_31	Phage portal protein	1672	2073
vO_31	Clp protease	2091	3230
vO_31	Major capsid protein gp17	3227	3946
vO_31	hp	3969	5129
vO_31	hp	5171	5670
vO_31	Head completion protein gp16	5657	6001
vO_31	hp	6001	6351
vO_31	hp	6348	6683
vO_31	Major tail protein	6687	7571
vO_31	hp	7624	7938
vO_31	Tape measure protein	8165	12064
vO_31	Phage tail protein	12105	12938
vO_31	hp	12951	13583
vO_31	Siphovirus ReqiPepy6 Gp37-like protein	13593	14024
vO_31	Tail fiber protein	13976	14914
vO_31	hp	14928	16706
vO_31	Tail fiber protein	16713	17234
vO_31	hp	17237	17824
vO_31	hp	17845	18357
vO_31	Bacteriophage holin family	18421	18858
vO_31	Peptidoglycan LD-endopeptidase CwlK	18830	19459
vO_31	hp	19524	19718
vO_31	Protein of unknown function (DUF3006)	19742	19966
vO_31	ComE operon protein 3	19963	20814
vO_31	Putative transcriptional regulator	20877	21092
vO_31	hp	21337	21540
vO_33	Pentapeptide repeats (8 copies)	2	496
vO_33	Helix-turn-helix	515	925
vO_33	Protein of unknown function (DUF739)	959	1183
vO_33	hp	1184	1549
vO_33	hp	1665	2285
vO_33	hp	2305	2622
vO_33	hp	2640	2882
vO_33	hp	2923	3312
vO_33	Lambda phage repressor protein cI	3461	3829
vO_33	hp	4017	4346
vO_33	hp	4361	4903
vO_33	Alphabaculovirus VLF1	4925	5968
vO_33	N-acetylmuramoyl-L-alanine amidase	6188	7108
vO_33	hp	7111	7344
vO_33	hp	7361	7735
vO_33	hp	7728	8039
vO_33	hp	8149	8325
vO_33	hp	8318	8431
vO_33	hp	8457	8708
vO_33	Reverse transcriptase	8705	9787
vO_33	hp	10164	12239
vO_33	hp	12259	12885
vO_33	Baseplate J-like protein	12882	13940
vO_33	hp	13940	14341
vO_33	Protein of unknown function (DUF2577)	14338	14739
vO_33	Tail tip assembly protein K	14732	15691
vO_33	LysM peptidoglycan-binding domain-containing protein	15704	16048
vO_33	Phage tape measure protein	16030	16227
vO_33	Phage XkdN-like tail assembly chaperone protein TAC	16227	18398
vO_33	Phage tail tube protein	18614	19045
vO_33	Tail sheath protein	19141	19617
vO_33	hp	19638	20156
vO_33	hp	20102	20935
vO_33	hp	20937	21143
vO_33	hp	21143	21589
vO_33	Head completion protein	21586	22155
vO_33	hp	22155	22505
vO_33	Major capsid protein	22499	22960

vO_33	Phage minor structural protein	22976	23269
vO_33	hp	23309	24358
vO_33	Minor head protein	24384	25016
vO_33	Phage portal protein	25170	25340
vO_33	Terminase large subunit	25340	27235
vO_33	hp	27225	28727
vO_33	hp	28817	30217
vO_33	Transcriptional activator rinA	30210	30716
vO_33	hp	30668	30847
vO_33	hp	30844	31260
vO_33	hp	31594	32166
vO_33	hp	32117	32668
vO_33	Thymidylate synthase	32665	32874
vO_33	hp	32979	33611
vO_33	DNA primase	33608	34168
vO_33	DNA polymerase	34189	36081
vO_33	hp	36119	37819
vO_33	hp	37932	38387
vO_33	AAA domain	38409	38747
vO_33	hp	38747	40387
vO_33	hp	40384	40800
vO_33	hp	40790	41407
vO_33	L-alanyl-D-glutamate peptidase	41530	41973
vO_33	DEAD box helicase	41966	42562
vO_33	hp	42534	44257
vO_34	hp	1	447
vO_34	Holin	456	668
vO_34	Terminase small subunit	665	925
vO_34	Spanin inner membrane subunit	938	1393
vO_34	Terminase large subunit	1390	3135
vO_34	hp	3354	3527
vO_34	hp	4300	4716
vO_34	hp	4713	4961
vO_34	hp	4958	5104
vO_34	hp	5175	5621
vO_34	hp	5689	6054
vO_34	hp	6143	6625
vO_34	hp	6622	6807
vO_34	hp	6804	7076
vO_34	hp	7076	10276
vO_34	RNA polymerase	10285	10458
vO_34	hp	10479	10916
vO_34	hp	10927	12057
vO_34	DNA ligase	12126	12593
vO_34	hp	12590	12712
vO_34	hp	12804	12998
vO_34	hp	12991	13653
vO_34	Deoxynucleotide monophosphate kinase	13650	13820
vO_34	Bacterial RNA polymerase inhibitor	13872	14579
vO_34	Helix-destabilizing protein	14579	15022
vO_34	Phage endonuclease I	15023	15463
vO_34	Endolysin	15537	16079
vO_34	hp	16066	16428
vO_34	hp	16437	18134
vO_34	Helicase	18146	18361
vO_34	hp	18426	18890
vO_34	hp	18899	20833
vO_34	DNA polymerase	20833	21225
vO_34	T7 Protein 5.3	21226	21663
vO_34	T7 Fusion protein 5.5/5.7	21660	21869
vO_34	hp	21866	22042
vO_34	hp	22039	22959
vO_34	Ribonuclease H	23113	23358
vO_34	Protein of unknown function (DUF2717)	23358	23630
vO_34	Family of unknown function (DUF5476)	23640	23906
vO_34	T3 Protein 7.3	23921	25537
vO_34	T7 Portal protein	25611	26516
vO_34	Capsid assembly scaffolding protein	26618	27646
vO_34	T7 Minor capsid protein	27706	28122

vO_34	T7 Tail tubular protein gp11	28098	28295
vO_34	T7 Tail tubular protein gp12	28306	30708
vO_34	T7 Probable scaffold protein gp13	30765	31202
vO_34	T7 Internal virion protein gp14	31215	31787
vO_34	T7 Internal virion protein gp15	31787	33985
vO_34	T7 Peptidoglycan transglycosylase gp16	34004	38116
vO_34	Phage tail fiber protein	38181	40136
vO_35	Uncharacterized protein YdhT	2	424
vO_35	hp	605	859
vO_35	hp	860	1054
vO_35	hp	1124	1426
vO_35	Repressor LexA	1449	2159
vO_35	Transcriptional repressor of cell division inhibition gene dicB	2227	2463
vO_35	hp	2467	2742
vO_35	hp	2817	3050
vO_35	hp	3446	4285
vO_35	hp	4282	4671
vO_35	hp	4668	5138
vO_35	hp	5135	5413
vO_35	hp	5410	5532
vO_35	hp	5529	5891
vO_35	hp	5991	6281
vO_35	HNH endonuclease	6268	6630
vO_35	hp	6630	6782
vO_35	hp	6779	7177
vO_35	Protein of unknown function (DUF1064)	7174	7578
vO_35	hp	7588	8067
vO_35	hp	8324	8551
vO_35	hp	8670	8840
vO_35	Putative nuclease YbcO	8833	9108
vO_35	hp	9108	9293
vO_35	Phage Lambda Protein ninB	9568	10059
vO_35	hp	10049	10366
vO_35	HNH endonuclease	10308	10589
vO_35	hp	10589	11119
vO_35	Terminase small subunit	11345	11596
vO_35	hp	11690	12091
vO_35	hp	12101	12271
vO_35	hp	12352	12585
vO_35	Phage Terminase	12630	14297
vO_35	Prohead protease	14333	14941
vO_35	Major capsid protein	14938	16278
vO_35	hp	16354	16533
vO_35	Phage portal protein	16544	17965
vO_35	hp	17962	18255
vO_35	hp	18263	18517
vO_35	Phage head-tail joining protein	18520	18867
vO_35	hp	18876	19337
vO_35	MIMIV putative nuclease	19435	20217
vO_35	Protein of unknown function (DUF3168)	20252	20611
vO_35	Tail fiber protein	20667	21131
vO_35	Phage tail assembly chaperone TAC	21171	21554
vO_35	hp	21638	21865
vO_35	hp	21991	22089
vO_35	Domain of unknown function (DUF4760)	22253	23503
vO_35	P22 antirepressor protein	23486	24127
vO_35	hp	24175	24558
vO_35	hp	24602	24922
vO_35	hp	24980	27076
vO_35	hp	27121	27624
vO_35	hp	27657	28049
vO_35	hp	28649	28804
vO_35	hp	28845	29999
vO_35	hp	29996	30883
vO_35	hp	30891	31124
vO_35	hp	31121	31312
vO_35	BspA type LRR	31296	34202
vO_35	hp	34202	34933
vO_35	hp	34999	35346

vO_35	Lysozyme	35346	35564
vO_35	hp	35554	36108
vO_35	Alphabaculovirus VLF1	36245	36469
vO_35	hp	36466	37686
vO_35	hp	37981	38184
vO_35	hp	38187	38465
vO_35	hp	38466	38711
vO_35	hp	38708	39166
vO_35	Exodeoxyribonuclease	39472	40083
vO_35	Essential recombination function protein	40070	40801
vO_35	hp	40811	40990
vO_35	hp	41004	41327
vO_35	hp	41324	42289
vO_35	hp	42364	42636
vO_35	Uncharacterized protein YdhT	42639	42890
vO_36	hp	1	120
vO_36	hp	599	886
vO_36	hp	883	1392
vO_36	hp	1408	1848
vO_36	hp	1841	2170
vO_36	hp	2791	3264
vO_36	hp	3279	3593
vO_36	Ribonuclease III	3647	3997
vO_36	hp	4001	4609
vO_36	hp	4609	6882
vO_36	hp	6882	7091
vO_36	hp	7088	7318
vO_36	hp	7337	8125
vO_36	hp	8122	9711
vO_36	hp	9708	10766
vO_36	hp	10787	12775
vO_36	hp	13123	13773
vO_36	hp	13767	13919
vO_36	hp	13949	14347
vO_36	SPbeta YomU	14350	15102
vO_36	hp	15109	15330
vO_36	hp	15334	15750
vO_36	hp	15776	16057
vO_36	Major capsid protein	16057	17061
vO_36	Phage Lambda head decoration protein	17103	17480
vO_36	Phage Lambda Capsid assembly protease	17487	18758
vO_36	Portal protein	18762	20243
vO_36	Head-tail connector	20244	20465
vO_36	Terminase large subunit	20493	22439
vO_36	hp	22436	23011
vO_36	hp	23125	23394
vO_36	hp	23484	23732
vO_36	Protein of unknown function (DUF3489)	23760	24344
vO_36	Modification methylase HpaI	24400	25563
vO_36	hp	25560	25700
vO_36	hp	25720	25917
vO_36	hp	26139	26747
vO_36	Endodeoxyribonuclease RusA	26926	27342
vO_36	hp	27342	27632
vO_36	hp	27833	28090
vO_36	hp	28087	28560
vO_36	hp	28557	29189
vO_36	hp	29900	30571
vO_36	Toxin-antitoxin system YdaS/YdaT	30564	30776
vO_36	hp	30773	31459
vO_36	hp	31786	32238
vO_36	hp	32300	32473
vO_36	hp	32470	32559
vO_36	hp	32556	32684
vO_36	RecT	32681	33571
vO_36	Exonuclease	33568	35430
vO_36	Single-strand DNA-binding protein	35430	35885
vO_36	Uncharacterized protein YkgJ	35895	36287
vO_36	hp	36342	36530

vO_36	hp	36527	36769
vO_36	Recombinase	36976	37545
vO_36	Transposase	37542	38741
vO_36	hp	38961	39230
vO_36	hp	39522	39746
vO_36	hp	39766	39996
vO_36	Modification methylase HpaI	40339	41859
vO_37	hp	47	352
vO_37	hp	382	1062
vO_37	Phage Lambda NinG	1059	1733
vO_37	Protein of unknown function (DUF1367)	1730	2317
vO_37	hp	2310	3059
vO_37	DNA replication protein DnaC	3061	3867
vO_37	hp	3857	4639
vO_37	hp	4639	4869
vO_37	Phage antirepressor protein KilA	4866	5672
vO_37	hp	5742	6164
vO_37	hp	6247	6441
vO_37	Transcriptional regulator DicA	6575	6862
vO_37	hp	6859	7680
vO_37	hp	7719	8522
vO_37	hp	8979	9233
vO_37	hp	9417	9821
vO_37	hp	9818	10009
vO_37	hp	10006	10200
vO_37	hp	10197	10322
vO_37	RecT	10479	11294
vO_37	Exonuclease	11291	12913
vO_37	hp	12910	13152
vO_37	hp	13149	13610
vO_37	hp	13692	14054
vO_37	hp	14056	14295
vO_37	hp	14404	15000
vO_37	hp	15088	15456
vO_37	Modification methylase DdeI	15510	17555
vO_37	hp	17556	17660
vO_37	hp	17804	18040
vO_37	hp	18131	18961
vO_37	hp	19024	19281
vO_37	hp	19430	19753
vO_37	hp	19750	20034
vO_37	hp	20108	20443
vO_37	hp	20493	20828
vO_37	hp	20825	21166
vO_37	hp	21217	21594
vO_37	hp	21641	22231
vO_37	hp	22559	23743
vO_37	Alphabaculovirus VLF1	24120	24446
vO_37	hp	24418	24942
vO_37	Spanin inner membrane subunit	24942	25364
vO_37	hp	25423	26145
vO_37	hp	26142	27347
vO_37	hp	27396	28274
vO_37	hp	28578	30710
vO_37	Baseplate hub protein	30853	32115
vO_37	Tail tip assembly protein I	32174	32788
vO_37	hp	32843	33199
vO_37	hp	33202	33528
vO_37	hp	33682	34167
vO_37	hp	34497	34817
vO_37	hp	34842	35606
vO_37	Tail tip assembly protein K	35608	36357
vO_37	Tail tip protein L	36367	36705
vO_37	Phage minor tail protein	36705	39755
vO_37	Tail length tape measure protein	39783	40088
vO_37	DUF1799	40106	40486
vO_37	Phage tail assembly chaperone	40496	41155
vO_37	Major tail protein	41195	41623
vO_37	Bacteriophage related domain of unknown function	41620	42216

vO_37	hp	42213	42581
vO_37	hp	42598	43101
vO_37	hp	43159	43734
vO_37	hp	43786	44754
vO_37	Major capsid protein	44766	45476
vO_37	hp	45564	45917
vO_37	Phage protein (N4 Gp49/phage Sf6 gene 66) family	45931	48291
vO_37	Minor head protein GP7	48288	49589
vO_37	Portal protein	49576	50037
vO_37	SPbeta YonF	50069	50692
vO_37	hp	50658	50900
vO_37	hp	50953	51270
vO_37	hp	51270	51641
vO_38	hp	2	100
vO_38	hp	97	297
vO_38	hp	342	704
vO_38	hp	817	1068
vO_38	hp	1071	1202
vO_38	hp	1208	1546
vO_38	hp	1606	2496
vO_38	hp	2656	3054
vO_38	hp	3063	3437
vO_38	hp	3827	4051
vO_38	hp	4494	4727
vO_38	hp	4738	4959
vO_38	hp	4959	5201
vO_38	DNA helicase	5579	7222
vO_38	hp	7223	7477
vO_38	hp	7474	7608
vO_38	hp	7609	7890
vO_38	RNA ligase	7890	9029
vO_38	hp	9031	9219
vO_38	hp	9374	9589
vO_38	hp	9860	10180
vO_38	hp	10387	10764
vO_38	hp	10736	10996
vO_38	Baseplate central spike complex protein	10986	11555
vO_38	Phage Lambda Tail fiber assembly protein	11569	11883
vO_38	hp	11886	12200
vO_38	hp	12200	13240
vO_38	hp	13253	14146
vO_38	hp	14146	14448
vO_38	Baseplate hub protein	14445	18161
vO_38	Tail tip assembly protein I	18174	18785
vO_38	Tail tip assembly protein K	18778	19581
vO_38	Tail tip protein L	19583	20329
vO_38	Minor tail protein	20339	20683
vO_38	Tail length tape measure protein	20773	24768
vO_38	hp	24752	24916
vO_38	Tail assembly chaperone	25081	25482
vO_38	Major tail protein	25559	26212
vO_38	Bacteriophage related domain of unknown function	26225	26701
vO_38	hp	26712	27110
vO_38	hp	27103	27495
vO_38	hp	27495	27899
vO_38	Major capsid protein	27958	29055
vO_38	hp	29120	29563
vO_38	Phage Lambda Capsid assembly protease C	29592	30920
vO_38	Mu-like prophage FluMu protein gp29	30924	32444
vO_38	Terminase large subunit	32457	33965
vO_38	hp	34045	34488
vO_38	hp	34501	34935
vO_38	hp	35416	35547
vO_38	hp	35818	36234
vO_38	hp	36640	36825
vO_38	Phage T4 protein in nrdA-td intergenic region	36888	37187
vO_38	hp	37175	37366
vO_38	hp	37350	37556
vO_38	hp	37553	37807

vO_38	RNA ligase	38220	39317
vO_38	hp	39314	39727
vO_38	Polynucleotide kinase	39724	40605
vO_38	hp	40615	40830
vO_38	Ribose-phosphate pyrophosphokinase	40827	41675
vO_38	MIMIV Uncharacterized HNH endonuclease	41653	42231
vO_38	Nicotinamide phosphoribosyltransferase	42234	44042
vO_38	GTP pyrophosphokinase	44145	44606
vO_38	hp	44599	44757
vO_38	hp	44754	44918
vO_38	hp	44915	45184
vO_38	hp	45181	45378
vO_38	hp	45371	45610
vO_38	hp	45607	45741
vO_38	hp	45738	45959
vO_38	hp	45949	46116
vO_38	hp	46116	46406
vO_38	hp	46403	46885
vO_38	hp	46889	47299
vO_38	Deoxycytidylate deaminase	47296	47715
vO_38	hp	47715	47921
vO_38	hp	47918	48100
vO_38	hp	48101	48367
vO_38	DNA ligase	48360	49580
vO_38	hp	49590	50117
vO_38	Serine/threonine protein phosphatase	50110	50856
vO_38	hp	50847	51017
vO_38	hp	51014	51208
vO_38	hp	51205	51588
vO_38	hp	51585	51773
vO_38	DNA primase	51955	52470
vO_38	hp	52467	52700
vO_38	hp	52882	53088
vO_38	hp	53069	53272
vO_38	hp	53266	53439
vO_38	hp	53511	53990
vO_38	hp	53987	54271
vO_38	hp	54271	54552
vO_38	hp	54540	55028
vO_38	hp	55182	55292
vO_38	hp	55279	55557
vO_38	hp	55561	55761
vO_38	hp	55867	56148
vO_38	hp	56135	56332
vO_38	hp	56333	56500
vO_38	hp	56484	56717
vO_38	hp	56677	56958
vO_38	hp	56955	57200
vO_38	hp	57197	57643
vO_38	hp	57840	58016
vO_38	hp	58013	58303
vO_38	hp	58300	58527
vO_38	hp	58520	58735
vO_38	hp	58803	59012
vO_38	hp	60098	60319
vO_38	hp	60330	60527
vO_38	hp	60538	60723
vO_38	hp	60734	60871
vO_38	hp	60868	61116
vO_39	Tail Protein	29	148
vO_39	Tail assembly protein	157	492
vO_39	Tail tube protein	540	1055
vO_39	major tail sheath protein	1113	2288
vO_39	hp	2407	2835
vO_39	Tail fibers protein	2839	4728
vO_39	Baseplate protein I	4725	5342
vO_39	Baseplate protein J	5342	6253
vO_39	Baseplate GP25	6250	6594
vO_39	Phage-baseplate injector	6591	7163

vO_39	hp	7239	8102
vO_39	Tail completion protein S	8150	8608
vO_39	Tail completion protein R	8598	9080
vO_39	Spanin inner membrane subunit	9177	9620
vO_39	hp	9617	9820
vO_39	Endolysin	9817	10662
vO_39	hp	10659	10937
vO_39	hp	10939	11292
vO_39	Tail Protein	11320	11532
vO_39	Head completion/stabilization protein	11532	11993
vO_39	Terminase	12098	12805
vO_39	Major capsid protein	12817	13824
vO_39	Scaffolding protein	13870	14748
vO_39	Terminase	14900	16657
vO_39	Portal protein	16657	17589
vO_40	hp	1	915
vO_40	hp	915	1805
vO_40	Tape measure protein	1809	4685
vO_40	hp	4698	4931
vO_40	hp	4906	5061
vO_40	hp	5061	5450
vO_40	hp	5477	5767
vO_40	hp	5773	6405
vO_40	hp	6410	6919
vO_40	hp	6935	7375
vO_40	hp	7368	7697
vO_40	Spbeta YomU	7701	8474
vO_40	Protein of unknown function DUF2513	8487	8723
vO_40	hp	8793	9215
vO_40	Protein of unknown function DUF1320	9215	9634
vO_40	hp	9638	9823
vO_40	Mu-like prophage major head subunit	9834	10739
vO_40	hp	10743	11021
vO_40	Mu-like prophage I protein	11014	12054
vO_40	hp	12397	12624
vO_40	hp	12663	13370
vO_40	hp	13354	13680
vO_40	Tail completion protein S	13776	14288
vO_40	Mu-like prophage FluMu F protein	14347	15660
vO_40	Uncharacterized protein YbiI	15641	15850
vO_40	Protein of unknown function DUF935	15843	17495
vO_40	hp	17507	18013
vO_40	Terminase large subunit	18016	19347
vO_40	hp	19376	19633
vO_40	Protein of unknown function (DUF3486)	19634	20161
vO_40	Mu phage uncharacterized protein gp26	20165	20488
vO_40	hp	20485	20778
vO_40	hp	20768	20992
vO_40	hp	20958	21263
vO_40	Lysozyme	21260	21442
vO_40	Mor transcription activator family	21439	21921
vO_40	hp	21968	22312
vO_40	hp	22309	22539
vO_40	Uncharacterized protein HI_1489	22536	22901
vO_40	Phage Mu GemA protein	22891	23334
vO_40	hp	23331	23771
vO_40	hp	23755	23961
vO_40	hp	23961	24491
vO_40	hp	24488	24688
vO_40	hp	24725	24964
vO_40	Target DNA activator MuB	24961	25182
vO_40	Transposase	25179	28130
vO_41	hp	313	633
vO_41	hp	644	1225
vO_41	hp	1301	3868
vO_41	hp	3888	4286
vO_41	Tail Protein	4283	5419
vO_41	Baseplate J-like protein	5412	6236
vO_41	Baseplate wedge protein	6233	6583

vO_41	hp	6699	6899
vO_41	Phage-baseplate injector	6919	7350
vO_41	Baseplate wedge protein	7343	8347
vO_41	Tail Protein	8335	8553
vO_41	Tape measure protein	8550	9455
vO_41	hp	9495	11822
vO_41	Tail assembly chaperone protein	12106	12351
vO_41	hp (DUF2513)	12348	12689
vO_41	Phage tail tube protein FII	12692	13216
vO_41	major tail sheath protein	13227	14441
vO_41	hp	14445	14633
vO_41	Minor tail protein U-like	14630	15112
vO_41	Protein of unknown function (DUF1320)	15114	15530
vO_41	hp	15534	15722
vO_41	Major capsid protein	15733	16647
vO_41	Mu-like prophage I protein	16651	17676
vO_41	hp	17941	18162
vO_41	Tail completion protein S	18191	18709
vO_41	Mu-like prophage FluMu F protein	18805	20133
vO_41	hp	20117	20323
vO_41	Protein of unknown function (DUF935)	20316	21923
vO_41	hp	21892	22371
vO_41	Terminase large subunit	22371	23702
vO_41	hp	23731	24021
vO_41	Protein of unknown function (DUF3486)	24022	24549
vO_41	Mu Phage gp26	24551	24877
vO_41	hp	24874	25161
vO_41	hp	25151	25711
vO_41	Lysozyme	25711	26196
vO_41	Mor transcription activator family	26242	26589
vO_41	Protein of unknown function (DUF1018)	26657	27100
vO_41	hp	27087	27278
vO_41	hp	27339	27812
vO_41	hp	27848	28096
vO_41	Target DNA activator MuB	28100	28876
vO_41	Transposase	28898	31081
vO_41	hp	31081	31269
vO_41	Repressor protein CI	31559	31825
vO_42	hp	174	491
vO_42	Polysaccharide deacetylase	493	2067
vO_42	hp	2099	2671
vO_42	hp	2874	3209
vO_42	hp	3211	3621
vO_42	hp	3636	3905
vO_42	hp	3909	4253
vO_42	hp	4294	5547
vO_42	hp	5663	6457
vO_42	Phage Terminase	6462	8186
vO_42	Portal protein	8183	9430
vO_42	Capsid protein	9588	11399
vO_42	Major tail protein	12027	12695
vO_42	hp	12738	13139
vO_42	hp	13208	13744
vO_42	hp	13775	14149
vO_42	Tape measure protein	14149	20199
vO_42	hp	20219	20980
vO_42	hp	20996	22864
vO_42	hp	22868	24286
vO_42	hp	24305	24619
vO_42	hp	24619	24768
vO_42	MIMIV Collagen-like protein 2	24805	26394
vO_42	SPbeta YorA	26414	28966
vO_42	hp	29017	29301
vO_42	N-acetylmuramoyl-L-alanine amidase	29304	30155
vO_42	Holin	30158	30385
vO_42	hp	30470	30703
vO_42	hp	30719	32548
vO_42	hp	32567	33172
vO_42	hp	33188	33391

vO_42	hp	33502	33717
vO_42	hp	33765	33965
vO_42	hp	34155	34250
vO_42	hp	34713	34988
vO_42	hp	35078	35185
vO_42	hp	35226	35558
vO_42	hp	35561	35677
vO_42	hp	35868	36170
vO_42	N-acetylmuramoyl-L-alanine amidase	36252	36884
vO_42	hp	36900	37097
vO_42	hp	37155	37463
vO_42	hp	37599	37973
vO_42	hp	37995	38240
vO_42	hp	38308	38472
vO_42	hp	38522	39229
vO_42	hp	39257	39616
vO_42	hp	39681	40511
vO_42	hp	40523	41143
vO_42	hp	41225	41572
vO_42	hp	41591	41923
vO_42	hp	41944	42348
vO_42	hp	42345	42749
vO_42	hp	42739	43737
vO_42	hp	43890	44045
vO_42	hp	44045	44236
vO_42	hp	44233	45003
vO_42	hp	44994	45161
vO_42	hp	45189	45740
vO_42	hp	45759	45920
vO_42	hp	46025	46432
vO_42	hp	46445	46600
vO_42	hp	46872	47051
vO_42	DNA polymerase/primase	47415	51314
vO_42	hp	51552	51923
vO_42	hp	52067	52519
vO_42	Helicase	52497	53672
vO_42	hp	53642	53968
vO_42	PD-(D/E)XK endonuclease-like domain	53934	54488
vO_42	hp	54503	57175
vO_42	hp	57177	57494
vO_43-44	hp	101	247
vO_43-44	hp	454	3000
vO_43-44	hp	3073	3345
vO_43-44	Spbeta YonP	3345	3524
vO_43-44	hp	3764	4045
vO_43-44	Tubulin-like protein CetZ	4045	5160
vO_43-44	Lysin A	5468	6076
vO_43-44	HTH-type transcriptional regulator	6251	6691
vO_43-44	hp	6804	6965
vO_43-44	hp	6968	7165
vO_43-44	hp	7122	7664
vO_43-44	hp	7727	8848
vO_43-44	hp	8862	8978
vO_43-44	FtsK/SpoIIIE protein	8968	11100
vO_43-44	hp	11106	11324
vO_43-44	Exodeoxyribonuclease recD	11529	13742
vO_43-44	hp	13745	14317
vO_45	hp	4	147
vO_45	hp	184	825
vO_45	hp	872	1396
vO_45	hp	1389	2012
vO_45	SPbeta YomG	2344	4776
vO_45	Spbeta Distal tail protein	4773	5549
vO_45	Soluble lytic murein transglycosylase	5614	8439
